# Supplementary material for: Male‐biased gene expression resolves sexual conflict through the evolution of sex‐specific genetic architecture
Source: Evol Lett. 2018 Feb 10;2(2):52–61. doi: 10.1002/evl3.39 (PMC6089503; doi:10.1002/evl3.39)
Supplement: Supplementary file 2 — Figure S1. Principal component analysis. Figure S2. Histogram of Tajima's D and FST across autosomal genes. Figure S3. Test for FST bias due to unequal coverage between sexes. Figure S4. Test for FST bias due to unequal coverage between sexes. Figure S5. Test for FST bias due to unequal coverage between sexes. Figure S6. Test for Tajima's D bias due to unequal coverage between sexes. Figure S7. Test for Tajima's D bias due to unequal coverage between sexes. Figure S8. Test for Tajima's D bias due to unequal coverage between sexes. [file EVL3-2-52-s002.docx]

**SUPPLEMENTARY MATERIALS & METHODS**

**Genome assembly**

We assembled a female *Poecilia reticulata* de novo genome based on two females from our outbred laboratory population, originally collected from the Quare River in Trinidad, and kept in closed, semi-natural captivity since 1998 ^1^. Illumina reads have been deposited in the NCBI Short Read Archive (PRJNA353986). DNA was sequenced on an Illumina HiSeq 4000 at The Wellcome Trust Centre for Human Genetics, University of Oxford and quality trimmed using Trimmomatic ^2^.

Detailed methods are presented elsewhere ^1^. Briefly, DNA-seq reads were error corrected with Quake v0.3.5 ^3^, and after trimming and filtering, roughly 480 million paired-end reads were assembled using SOAPdenovo v2.04 ^4^, resulting in on average 150X genome coverage. Scaffolds were assigned a positional location by BLASTing ^5^ guppy genes from RefSeq ^6^ to the de novo assembly. Genomic coverage and polymorphism analyses were used in combination to distinguish *P. reticulata* sex chromosomes from the autosomes ^1^.

**Investigating population structure**

We calculated genotype posterior probabilities at each site for each individual from ANGSD using a prior information under the assumption of Hardy Weinberg Equilibrium.

We next used ngsPopGen ^7^ in ngsTools ^8^ to estimate the covariance matrix between individuals based on genotype probabilities. Finally, we performed an eigenvector decomposition and plotted the first and second component of the PCA.

**Calculating inbreeding coefficients**

We estimated allele frequencies with ANGSD ^9^ using the SAMtools genotype likelihood model and assuming the reference base being one of the two possible alleles ^9^. Triallelic sites or sites with SNP pvalue > 1e-6 were removed. We estimated pairwise linkage disequilibrium from unphased expected genotypes calculated from genotype likelihoods. We then pruned linked sites using a threshold on r^2^ of 0.3 and retrieved 86036 unlinked sites for further analysis.

Inbreeding coefficients were calculated using an EM algorithm with the ngsF package ^10^ in ngsTools ^8^. The maximum root-mean-square deviation between iterations to assume convergence was 0.001.

**Test for bias in estimating summary statistics due to unequal coverage between sexes**

To test whether uneven depth between males and females, due to sex-biased expression, biases the estimation of per-gene Tajima’s D or F_ST_ between sexes, we simulated genomes for two populations using ms ^11^. Specifically, we simulated two populations of equal and constant size diverging without gene flow 20, 200 and 2,000 generations ago. The same filtering criteria used for the F_ST_ and Tajima’s D analysis was imposed. We assumed an effective population size of 1000 and mutation and recombination rates of 5*10^(-8) per base per generation ^6^. Under this model we simulated genes of 25kbp each, and a pseudo-chromosome of 10Mbp. We simulated 10,000 genes for each scenario. Sample sizes of the populations were set to 11 and 4 diploid individuals, representing male and female samples in our study respectively. From each simulated gene and chromosome, we simulated sequencing reads at the polymorphic sites using msToGlf from ANGSD ^9^. We simulated three different sequencing scenarios with both sexes at high mean depth (30X), males at low depth (5X) and females at high depth (30X), males at high depth (30X) and females at low depth (5X). We then used the previously described pipeline to estimate F_ST_ between sexes and Tajima’s D for the whole population. For each scenario, Kendall's tau coefficient was used to assess the correlation between estimates of summary statistics. Kendall's tau measures association using a rank-based method accounting for ties.

**Power assessment for detecting outliers of summary statistics due to unequal and low sample size between sexes**

We simulated data with ms ^11^ to assess the power to detect outlier genes based on F_ST_ and Tajima’s D in case of high and even sample size compared to our data set with unequal and low sample size. For consistency with previous analyses, we assumed an ancestral effective population size of 1,000, mutation and recombination rates of 5*10^(-8) per base per generation, and an average gene length of 25kbp. We simulated sets of 1,000 genes along with a pseudo-chromosome of 10Mbp used to derive prior information.

In the case of F_ST_, we simulated 950 genes with a divergence time between the two populations of 40 generations. We then simulated 50 genes with no population structure and assessed the power to identify these outliers within the lowest 5^th^ percentile of the F_ST_ distribution. Similarly but separately, we simulated 50 genes with a deeper divergence time (400 generations ago) and assessed the power to identify these outliers within the highest 95^th^ percentile of the F_ST_ distribution.

In the case of Tajima’s D, we simulated 950 genes with an effective population size of 10,000. We then simulated 50 genes with an effective population size of 100,000 and assessed the power to identify these outliers within the lowest 5^th^ percentile of the Tajima’s D distribution. Similarly but separately, we simulated 50 genes with a lower effective population size (1,000) and assessed the power to identify these outliers within the highest 95^th^ percentile of the TD distribution.

We use the Matthews correlation coefficient ^13^ to quantify the accuracy of the classification between outliers and non-outliers based on F_ST_ and Tajima’s D distributions. This coefficient is calculated from the classic confusion matrix. A value of 0 is indicative of random prediction while positive values show agreement between predictions and observations. This metric is related to the classic F1-score but it additionally takes false negative rates into account.

We calculated Matthews correlation coefficient for detecting outliers in F_ST_ and Tajima’s D distribution for both large and even sample sizes (40 individuals in total) and small and uneven sample sizes (4 and 11 individuals per population).

**Test for bias in identifying sex-biased genes due to unequal number of samples between sexes**

Male guppies show a remarkable variety of colouration patterns in the wild and our male samples exhibit high phenotypic diversity. Therefore, we predict higher variability in expression across males than females, which can in turn influence the identification of sex-biased genes. We chose to use more male individuals (eleven samples) than females (four samples) to mitigate this and ensure the full transcriptional variation seen in males was reflected in this study. However, to exclude the possibility that the unequal number of male and female samples has biased the identification of sex-biased genes, and therefore measures of Tajima’s D or F_ST_ across sets of genes, we conducted pairwise rank order correlations of autosomal expression across samples. We calculated pairwise Spearman’s rank correlation across female samples, and calculated average rho and p-value using the R ^12^ package Hmisc. 95% confidence intervals were calculated using bootstrapping with 1000 replicates. We repeated this for male samples. Finally, we sampled four males at random, 1000 times, and calculated average rho.

**SUPPLEMENTARY RESULTS**

We performed population genetic analyses using RNA-seq data and the guppy genome that we previously assembled and annotated ^1^. Briefly, we mapped RNA-seq data from male and female tails (S1 Table) ^14^ in order to estimate allele frequencies and genotype likelihoods using ANGSD ^9^ and ngsTools ^8^. ANGSD uses an empirical Bayes approach to account for the uncertainty in genotype calling from next-generation sequencing data when estimating neutrality test statistics. Estimates of site frequency spectra from next-generation data can be heavily biased by differences in coverage in next-generation sequencing data and ANGSD circumvents this problem by directly analyzing genotype likelihoods ^9^. This approach has obvious benefits for our analyses given the large variance in coverage associated with expression data.

**Population structure**

We performed a Principal Component Analysis on the genotype likelihoods of male and female guppies to investigate population structure. In particular, population structure between male and female individuals could confound our estimation of Tajima’s D and associated statistics. The first principal component explains 9.47% of the variance across individuals and the second component 8.8% (S1 Figure). We repeated the analysis after excluding sites on the sex chromosomes, which are subject to distinct evolutionary pressures relative to the autosomes ^15^, and found similar patterns (S1 Figure). We conclude there is no genetic population structure between males and females in our analysis, consistent with a single large, outbred laboratory population

We estimated Tajima’s D for all genes within the guppy genome. For autosomal genes with *Poecilia formsa* orthologs (9582 genes, Sign test p<0.001, median=0.609), median Tajima’s D is significantly greater than 0 (S2 Figure, Panel A), indicating a population contraction, consistent with initial collections used to establish the captive laboratory population.

**Inbreeding coefficients**

We also calculated inbreeding coefficients across our population using unlinked sites. Mean inbreeding coefficient across all individuals is 0.074 and we therefore assume Hardy Weinberg equilibrium for subsequent analyses. To further test for deviation from Hardy Weinberg Equilibrium, we calculated the likelihood of all samples being inbred, with inbreeding coefficients varying from 0.1 to 0.2. Our null hypothesis is that each sample has its estimated inbreeding coefficient while the alternate hypothesis is that all samples are highly inbred. A likelihood ratio test largely fails to reject the null hypothesis (p-value < 0.001, chi-square test, d.f=15) suggesting that all samples are either non-inbred or exhibit a negligible inbreeding coefficient.

**Test for bias in estimating summary statistics due to unequal coverage between sexes**

Based on simulated data, we observed no bias estimating inter-sexual F_ST_ for populations with uneven depth compared to populations with equal high depth both for recent (Kendall’s tau=0.85/Spearman’s rho=0.95 for low-depth males and tau=0.78/rho=0.91 for low-depth females) (Figure S3), medium (Kendall’s tau=0.87/ Spearman’s rho=0.96 for low-depth males and tau=0.81/rho=0.94 for low-depth females) (Figure S4) and old divergence (Kendall’s tau=0.91/Spearman’s rho=0.97 for low-depth males and tau=0.88/rho=0.96 for low-depth females) (Figure S5). Likewise, we did not observe any significant bias in estimating Tajima’s D at the whole population with uneven depth compared to populations with equal high depth both for recent (Kendall’s tau=0.94/Spearman’s rho=0.99 for low-depth males and tau=0.96/rho=1.00 for low-depth females) (Figure S6), medium (Kendall’s tau=0.93/Spearman’s rho=0.99 for low-depth males and tau=0.96/rho=1.00 for low-depth females) (Figure S7) and old divergence (Kendall’s tau=0.92/Spearman’s rho=0.99 for low-depth males and tau=0.91/rho=0.99 for low-depth females) (Figure S8). All p-values for these correlations are highly significant (p<0.0001, Kendall's rank correlation tau test).

**Power assessment for detecting outliers of summary statistics due to unequal and low sample size between sexes**

Based on simulated data, we observe no notable decrease in Matthews correlation coefficient between cases of large/even and small/uneven sample sizes for detecting low F_ST_ values (0.50 and 0.31, respectively), high F_ST_ values (0.83 and 0.83, respectively), low Tajima’s D values (0.96 and 0.88, respectively), high Tajima’s D values (0.92 and 0.62, respectively). Although here we consider only a single scenario for generating outliers, we show that our experimental set up of low and uneven sample size is able to generate reliable predictions of outliers based on positive values for the Matthews correlation coefficient.

**Test for bias in identifying sex-biased genes due to unequal number of samples between sexes**

We found significant (p<0.001) Spearman’s rank correlation across all pairwise female comparisons with an average rho of 0.935 (95% CI = 0.936-0.981). We found significant (p<0.001) Spearman’s rank correlation across all pairwise male comparisons with an average rho of 0.919 (95% CI = 0.917-0.939). Although marginally non-significant, the lower correlation across male samples relative to females is consistent with higher phenotypic diversity in the tail due to a high variation in colour patterns. Furthermore, when we re-sampled males with 1000 repetitions, we found a similarly high correlation across four randomly sampled individuals, with an average rho of 0.919. Together, this suggests that our statistical power to identify sex-biased genes is not limited by the unbalanced sample number.

**S1 Table**: RNA-seq information for each sample ^1^.

| **Sample** | **Raw paired reads** | **Paired reads after trimming** | **% removed** |
| --- | --- | --- | --- |
| Female_1 | 31,614,139 | 30,942,700 | 2.12 |
| Female_4 | 42,162,172 | 41,258,427 | 2.14 |
| Female_7 | 29,059,783 | 28,520,214 | 1.86 |
| Female_13 | 32,392,057 | 31,628,760 | 2.36 |
| Male_5 | 26,029,449 | 25,434,019 | 2.29 |
| Male_8 | 31,955,977 | 31,182,426 | 2.42 |
| Male_11 | 36,271,770 | 35,430,424 | 2.32 |
| Male_17 | 30,579,016 | 29,853,906 | 2.37 |
| Male_6 | 31,303,333 | 30,559,027 | 2.38 |
| Male_9 | 37,546,710 | 36,718,647 | 2.21 |
| Male_15 | 29,875,402 | 29,258,994 | 2.06 |
| Male_18 | 39,603,241 | 38,785,961 | 2.06 |
| Male_12 | 32,568,129 | 31,793,985 | 2.38 |
| Male_2 | 23,196,956 | 22,777,671 | 1.81 |
| Male_14 | 31,859,225 | 31,110,939 | 2.35 |

| **Model** | **Δ AIC**  **(AIC)** | **LogLikelihood**  **(LRT p-value)*** |
| --- | --- | --- |
| **Intercept** | 8.84  (21450.50) | -10723 (p< 0.001) |
| **Linear** | -  (21441.65) | -10718  - |
| **2nd degree polynomial** | 1.96  (21443.61) | -10718 (p=0.839) |
| **3rd degree polynomial** | 2.28  (21443.61) | -10717  (p= 0.424) |
| **4th degree polynomial** | 4.01  (21445.66) | -10717  (p= 0.574) |
| **Best fit, adjusted r^2^** | Linear, 0.001 | |

**S2 Table:** Model selection for relationship between Tajima’s D and sex-bias for autosomal genes**.**

*Significance test relative to the model with the lowest AIC. A significant p-value indicates the model with the lowest AIC is the better fitting model. Models within 2 AIC units of the model with the lowest AIC (or p <0.05) were treated as one top model set, and the model with the fewest parameters was preferred.

**S3 Table:** Model selection for relationship between Tajima’s D and sex-bias for autosomal genes excluding immunity genes.

| **Model** | **Δ AIC**  **(AIC)** | **LogLikelihood**  **(LRT p-value)*** |
| --- | --- | --- |
| **Intercept** | 11.45  (21278.67) | -10637 (p< 0.001) |
| **Linear** | -  (21267.23) | -10631  - |
| **2nd degree polynomial** | 1.91  (21269.14) | -10631 (p=0.763) |
| **3rd degree polynomial** | 1.66  (21268.89) | -10629  (p= 0.311) |
| **4th degree polynomial** | 3.55  (21270.78) | -10629  (p= 0.484) |
| **Best fit, adjusted r^2^** | Linear, 0.001 | |

*Significance test relative to the model with the lowest AIC. A significant p-value indicates the model with the lowest AIC is the better fitting model. Models within 2 AIC units of the model with the lowest AIC (or p <0.05) were treated as one top model set, and the model with the fewest parameters was preferred.

**S4 Table:** Model selection for relationship between F_ST_ and sex-bias for autosomal genes.

| **Model** | **Δ AIC** | **LogLikelihood**  **(LRT p-value)*** |
| --- | --- | --- |
| **Intercept** | 3.54  (-22028.24) | 11016 (p= 0.021) |
| **Linear** | 4.94  (-22026.84) | 11016  (p= 0.012) |
| **2nd degree polynomial** | 0.52  (-22031.26) | 11020 (p= 0.104) |
| **3rd degree polynomial** | 1.76  (-22030.02) | 11020  (p= 0.053) |
| **4th degree polynomial** | -  (-22031.78) | 11022  - |
| **Best fit, adjusted r^2^** | 2^nd^ degree polynomial, <0.001 | |

*Significance test relative to the model with the lowest AIC. A significant p-value indicates the model with the lowest AIC is the better fitting model. Models within 2 AIC units of the model with the lowest AIC (or p <0.05) were treated as one top model set, and the model with the fewest parameters was preferred.

**S5 Table:** Model selection for relationship between F_ST_ and sex-bias for autosomal genes excluding immunity genes.

| **Model** | **Δ AIC** | **LogLikelihood**  **(LRT p-value)*** |
| --- | --- | --- |
| **Intercept** | 3.64  (-21868.00) | 10936 (p= 0.020) |
| **Linear** | 4.73  (-21866.91) | 10936  (p= 0.013) |
| **2nd degree polynomial** | 0.35  (-21871.29) | 10940 (p= 0.114) |
| **3rd degree polynomial** | 1.72  (-21869.92) | 10940  (p= 0.053) |
| **4th degree polynomial** | -  (-21871.64) | 10942  - |
| **Best fit, adjusted r^2^** | 2^nd^ degree polynomial, <0.001 | |

*Significance test relative to the model with the lowest AIC. A significant p-value indicates the model with the lowest AIC is the better fitting model. Models within 2 AIC units of the model with the lowest AIC (or p <0.05) were treated as one top model set, and the model with the fewest parameters was preferred.

**S6 Table:** Median values of F_ST_ and Tajima’s D for autosomal genes excluding immunity genes.

|  | **Male-biased**  p-value | **Female-biased**  p-value | **Unbiased** |
| --- | --- | --- | --- |
| Tajima’s D | 0.515  p=0.011 | 0.571  p=0.827 | 0.614 |
| Inter-sexual F_ST_ | 0.026  p=0.019 | 0.032  p=0.026 | 0.016 |

We used standard fold-change thresholds to define female-biased (log_2_ male:female RPKM < -1) and male-biased (log_2_ male:female RPKM > 1). Wilcoxon test relative to unbiased genes.

**S7 Table:** Distinguishing scenarios of the types of sexual conflict acting across the autosomes.

| **Scenario** | **Pattern** | **Sex-biased**  Obs/Exp  p-value | **Male-biased**  Obs/Exp  p-value | **Female-biased**  Obs/Exp  p-value | **Unbiased**  Obs/Exp  p-value |
| --- | --- | --- | --- | --- | --- |
| **I.** | Sexual conflict due to differences in reproductive fitness | 34/45  p=0.110 | 19/28  p=0.100 | 15/17  p=0.580 | 1070/1059  p=0.740 |
| **II.** | Sexual conflict due to differences in viability selection | 54/48  p=0.390 | 30/29  p=0.920 | 24/19  p=0.200 | 1128/1134  p=0.860 |
| **III.** | Sex-specific viability effects | **54/39**  **p=0.020** | 32/24  p=0.110 | 22/15  p=0.08 | 915/930  p=0.630 |

Female-biased genes are defined as genes with log_2_ fold change < -1 and significant p-value, male-biased genes are defined as genes with log_2_ fold change > 1 and significant p-value. High Tajima’s D was defined as > 0.893 and low Tajima’s D was defined as < 0.272 to account for the inferred population contraction within our population (Supporting Results). High F_ST_ was defined as > 0.047 and low F_ST_ was defined as < -0.008 (Supporting Results).

**S1 Figure.** Principal component analysis. Female individuals are in red and males are in blue. Panel A. PCA conducted using all genes. Panel B. PCA conducted excluding genes on the sex chromosomes.

**S2 Figure**. Histogram of Tajima’s D and F_ST_ across autosomal genes. Dotted line indicates median value and *** indicates significant difference from 0 (Sign Test ; p<0.001). Panel A. Distribution of Tajima’s D for autosomal genes with *Poecilia formosa* reciprocal orthologs after removal of immunity genes. Panel B. Distribution of inter-sexual F_ST_ for autosomal genes with *Poecilia formosa* reciprocal orthologs after removal of immunity genes*.*


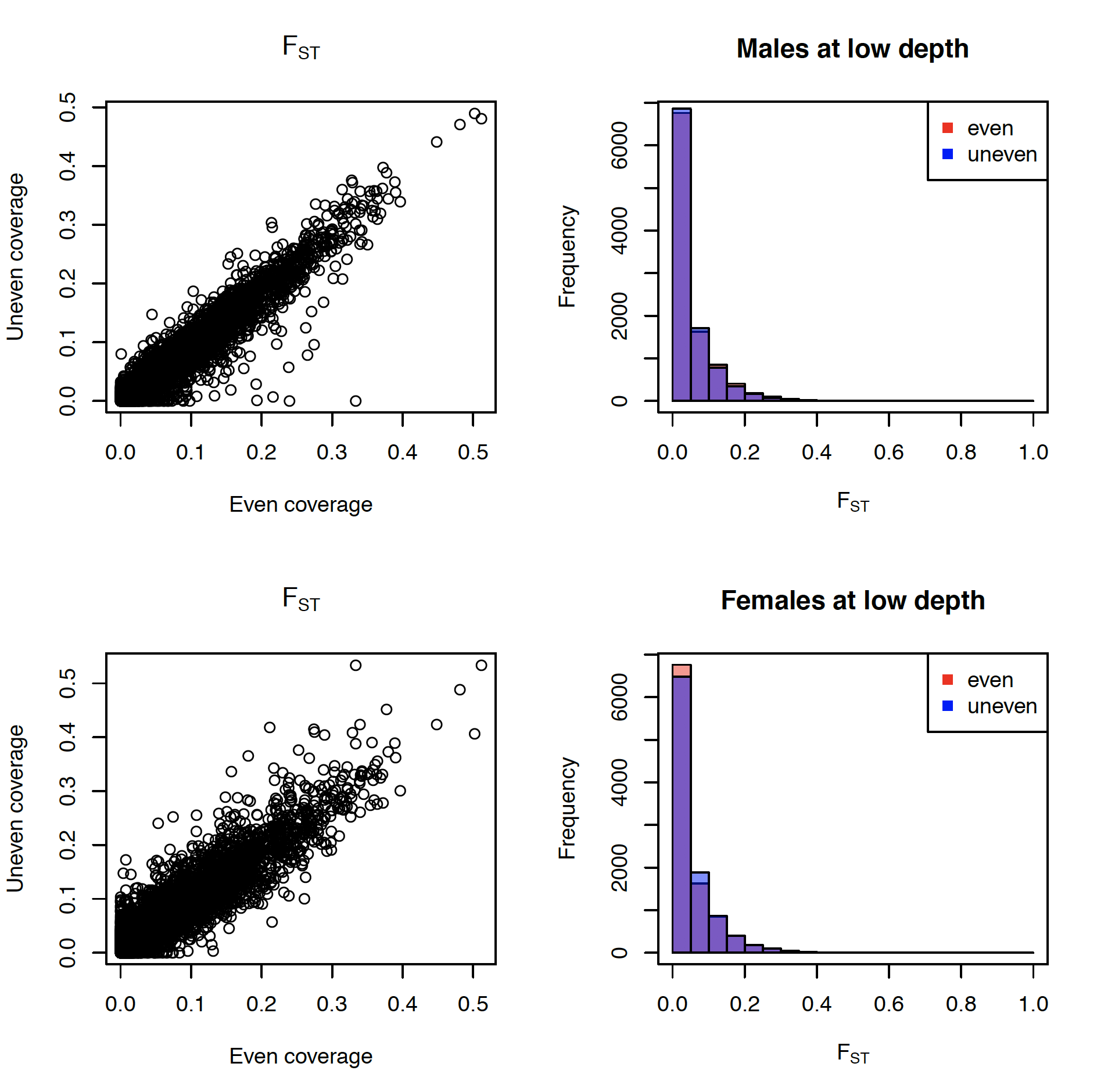


**S3 Figure**. Test for F_ST_ bias due to unequal coverage between sexes. Upper panel. Correlation (left panels) between inter-sexes F_ST_ values calculated from even high coverage data and values estimated from uneven coverage, under a scenario of recent divergence and male (upper panel) or female (lower panels) samples at low depth. Histograms of the two distributions are presented on the right panels.


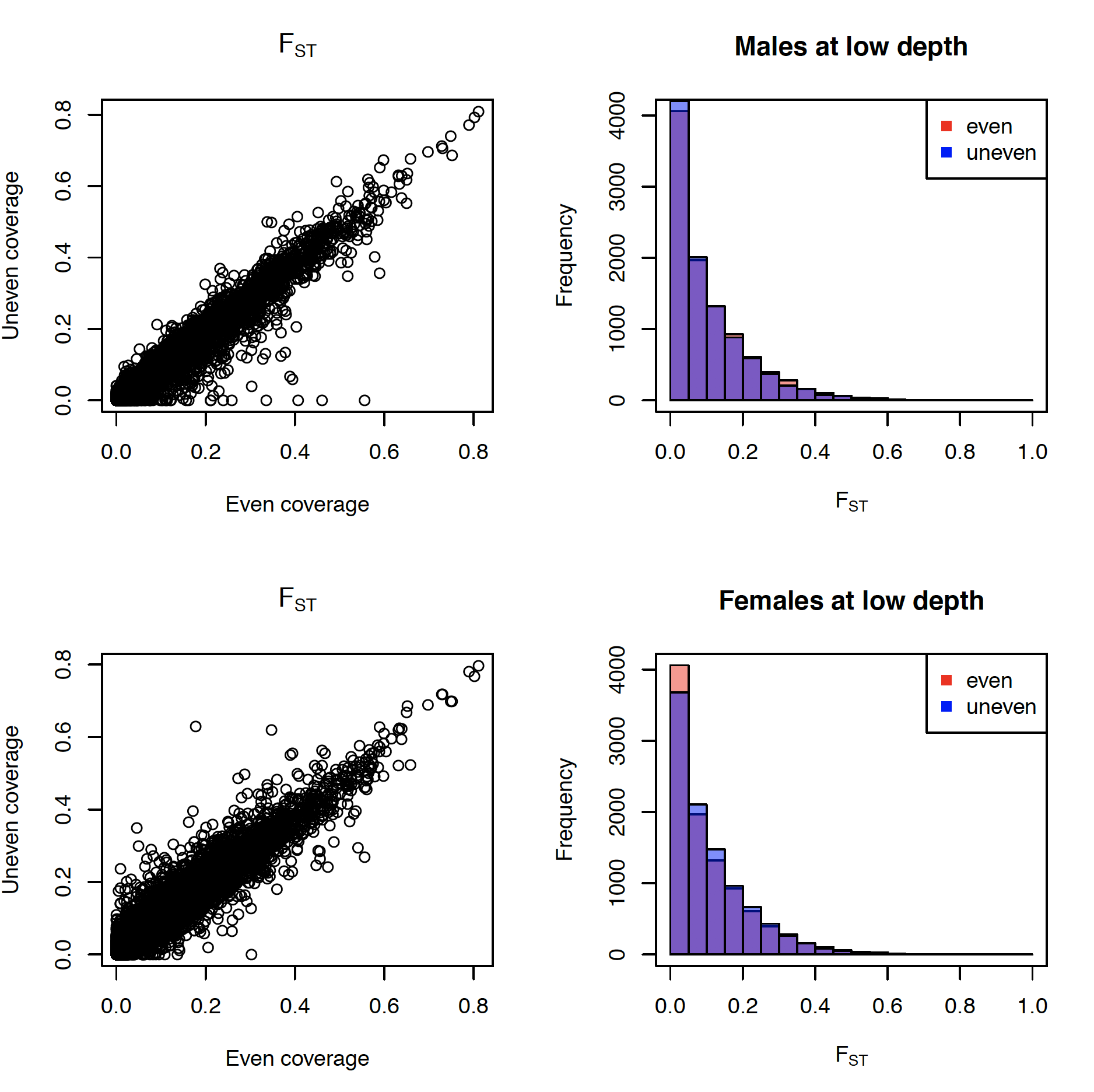


**S4 Figure**. Test for F_ST_ bias due to unequal coverage between sexes. Upper panel. Correlation (left panels) between inter-sexes F_ST_ values calculated from even high coverage data and values estimated from uneven coverage, under a scenario of medium divergence and male (upper panel) or female (lower panels) samples at low depth. Histograms of the two distributions are presented on the right panels.

**
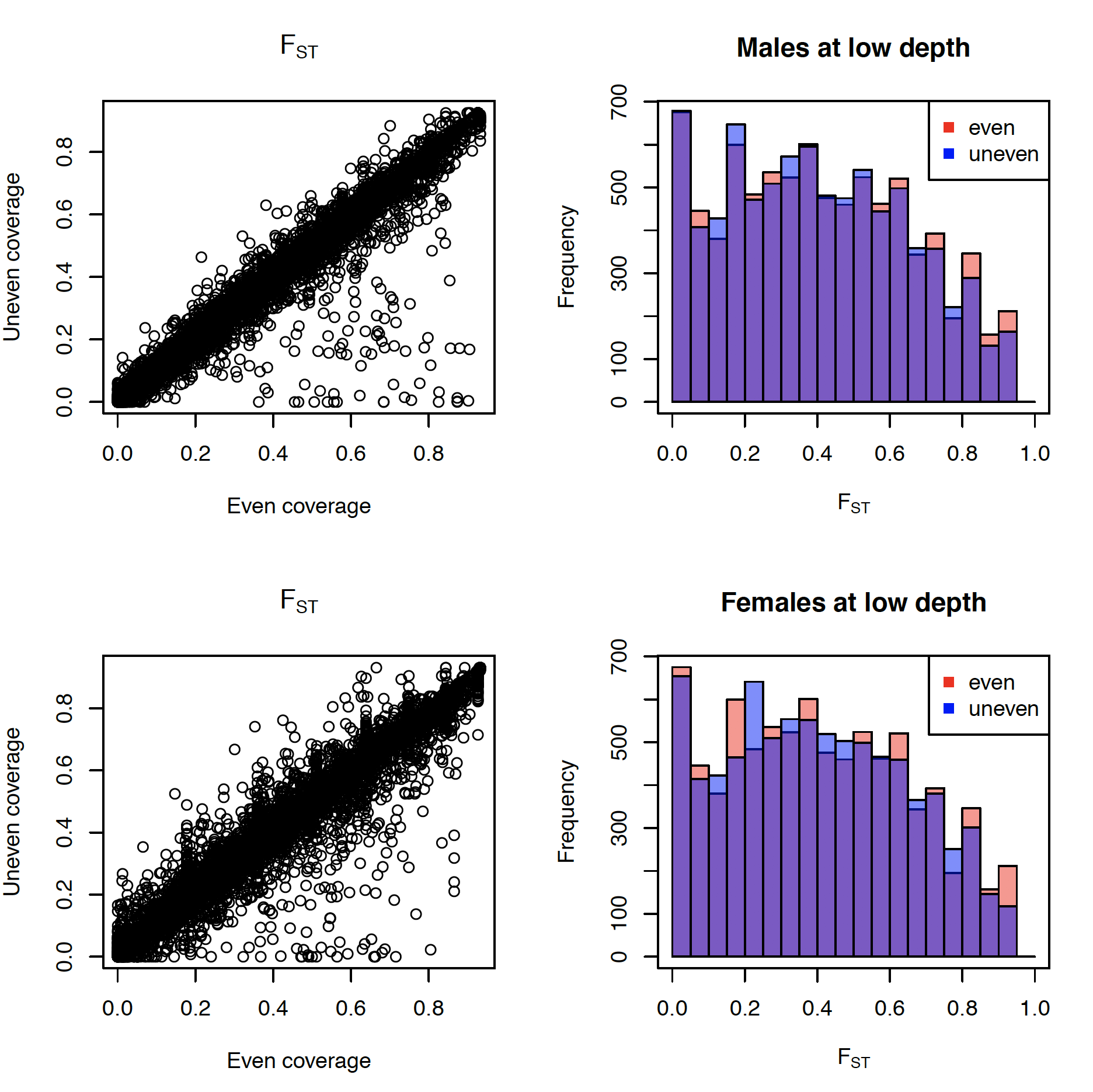
**

**S5 Figure**. Test for F_ST_ bias due to unequal coverage between sexes. Upper panel. Correlation (left panels) between inter-sexes F_ST_ values calculated from even high coverage data and values estimated from uneven coverage, under a scenario of medium divergence and male (upper panel) or female (lower panels) samples at low depth. Histograms of the two distributions are presented on the right panels.

**S6 Figure**. Test for Tajima’s D bias due to unequal coverage between sexes. Upper panel. Correlation (left panels) between Tajima’s D values calculated from even high coverage data and values estimated from uneven coverage, under a scenario of recent divergence and male (upper panel) or female (lower panels) samples at low depth. Histograms of the two distributions are presented on the right panels.

**S7 Figure**. Test for Tajima’s D bias due to unequal coverage between sexes. Upper panel. Correlation (left panels) between Tajima’s D values calculated from even high coverage data and values estimated from uneven coverage, under a scenario of medium divergence and male (upper panel) or female (lower panels) samples at low depth. Histograms of the two distributions are presented on the right panels.

**S8 Figure**. Test for Tajima’s D bias due to unequal coverage between sexes. Upper panel. Correlation (left panels) between Tajima’s D values calculated from even high coverage data and values estimated from uneven coverage, under a scenario of old divergence and male (upper panel) or female (lower panels) samples at low depth. Histograms of the two distributions are presented on the right panels.

**REFERENCES**

1. Wright, A.E. *et al.* Convergent recombination suppression suggests role of sexual selection in guppy sex chromosome formation. *Nature Communications* **8**, 14251 (2017).

2. Lohse, M. *et al.* RobiNA: a user-friendly, integrated software solution for RNA-Seq-based transcriptomics. *Nucleic Acids Research* **40**, W622-W627 (2012).

3. Kelley, D.R., Schatz, M.C. & Salzberg, S.L. Quake: quality-aware detection and correction of sequencing errors. *Genome Biology* **11**(2010).

4. Luo, R.B. *et al.* SOAPdenovo2: an empirically improved memory-efficient short-read de novo assembler. *Gigascience* **1**(2012).

5. Altschul, S.F., Gish, W., Miller, W., Myers, E.W. & Lipman, D.J. Basic local alignment search tool. *Journal of Molecular Biology* **215**, 403-410 (1990).

6. Künstner, A. *et al.* The genome of the Trinidadian guppy, Poecilia reticulata, and variation in the Guanapo population. *PLOS ONE* **11**, e0169087 (2016).

7. Fumagalli, M. *et al.* Quantifying population genetic differentiation from next-generation sequencing data. *Genetics* **195**, 979-992 (2013).

8. Fumagalli, M., Vieira, F.G., Linderoth, T. & Nielsen, R. ngsTools: methods for population genetics analyses from next-generation sequencing data. *Bioinformatics* **30**, 1486-1487 (2014).

9. Korneliussen, T.S., Albrechtsen, A. & Nielsen, R. ANGSD: Analysis of next generation sequencing data. *BMC Bioinformatics* **15**(2014).

10. Vieira, F.G., Fumagalli, M., Albrechtsen, A. & Nielsen, R. Estimating inbreeding coefficients from NGS data: Impact on genotype calling and allele frequency estimation. *Genome Research* **23**, 1852-1861 (2013).

11. Hudson, R.R. Generating samples under a Wright-Fisher neutral model of genetic variation. *Bioinformatics* **18**, 337-8 (2002).

12. *R: A Language and Environment for Statistical Computing. R Foundation for Statistical Computing*, (R version 2.15.2, Vienna, Austria, 2012).

13. Matthews, B.W. Comparison of the predicted and observed secondary structure of T4 phage lysozyme. *Biochimica et Biophysica Acta (BBA) - Protein Structure* **405**, 442-451 (1975).

14. Kotrschal, A. *et al.* The benefit of evolving a larger brain: big-brained guppies perform better in a cognitive task. *Animal Behaviour* **86**, e4-e6 (2013).

15. Wright, A.E. & Mank, J.E. The scope and strength of sex-specific selection in genome evolution. *Journal of Evolutionary Biology* **26**, 1841-1853 (2013).
